# Dietary selenium supplementation: its impact on growth, microbiota, antioxidant capacity, and immunity in Inner Mongolian cashmere goats

**DOI:** 10.3389/fvets.2025.1650280

**Published:** 2025-10-14

**Authors:** Yuliang Wen, Yuping Xiang, Yajing Shao, Tao Wang, Jianyong Liang, Bin Liu, Tiecheng Wu

**Affiliations:** ^1^College of Animal Science and Veterinary Medicine, Henan Institute of Science and Technology, Xinxiang, China; ^2^Inner Mongolia Academy of Agricultural and Animal Husbandry Sciences, Hohhot, China

**Keywords:** yeast selenium, antioxidant, rumen microorganisms, cashmere goat, immunity

## Abstract

Selenium (Se) is an essential trace element in the animal body that plays a crucial role in regulating antioxidant defense, immune response, and reproductive performance, contributing significantly to overall animal health. This study aimed to investigate the effects of dietary yeast selenium added to the feed on growth performance, antioxidant capacity, immune response, and rumen microbes of Inner Mongolian cashmere goats. A total of 18 male Inner Mongolian cashmere goats (47.1 ± 1.1 kg, 24 months of age) were selected and randomly divided into two groups, the normal feeding group (CON group) and the yeast selenium supplementation group (0.4 mg/d, Se group) (*n* = 9 in each group) for 30 days of the experiment. The results showed that the average daily weight gain in the Se group was significantly higher than that in the CON group (*p* < 0.05). In addition, the levels of glutathione peroxidase (GSH-Px), superoxide dismutase (SOD), immunoglobulin IgA, and IgG in the blood and semen in the Se group were significantly higher than those in the CON group (*p* < 0.05). Dietary supplementation with yeast selenium mainly increased the abundance of *Bacteroidales_RF16_group*, *p-251-o5*, *UCG-004*, and *UCG-010* in the rumen (*p* < 0.05). Spearman’s correlation analysis showed that *Bacteroidales_RF16_group*, *Bacteroidales_RCG-004*, and *Bacteroidales_RCG-010* significantly affected the rumen microbiota and fermentation parameters and significantly increased the antioxidant and immune capacity of plasma and seminal plasma (*p* < 0.05). This study provides data to support the use of supplemental yeast selenium for the healthy breeding of cashmere goats.

## Introduction

The Inner Mongolia white cashmere goat is considered one of the world’s highest-quality cashmere-producing breeds, and its cashmere is a valuable raw material for textiles, often referred to as the “fiber gem” or “soft gold.” It is regarded as the premium choice for high-end international luxury brands (1). However, this breed faces challenges in maintaining optimal health and productivity because of environmental stressors and nutritional deficiencies.

Selenium (Se) plays a vital role in the growth, development, and physiological homeostasis of both animals and humans (2, 3). Dietary selenium deficiency often leads to a range of health issues in livestock, including reduced fertility, retained placenta, and increased incidence of mastitis and uterine inflammation in cattle (4), mulberry heart disease in pigs (5), and cardiac damage in poultry (6). Oxidative stress, caused by metabolic processes and environmental factors, can disrupt cellular homeostasis, particularly in reproductive tissues. For example, supplementation with 1.0 μg/mL Se-NP-enriched semen enhancer improves sperm quality in thawed Holstein bull semen and increases fertilization rates by reducing apoptosis, lipid peroxidation, and cryopreservation-induced damage (7). In female goats, selenium modulates reproductive hormones, including estrogen (E2), progesterone (P4), and follicle-stimulating hormone (FSH), which are critical for fertilization and embryonic development (8). In addition, selenium exerts significant immunomodulatory effects. Selenium supplementation has been shown to elevate immunoglobulin (IgA, IgM, and IgG) concentrations and cytokine levels (IL-2, IL-6, and IL-10) (9, 10). Dietary supplementation with 0.375 mg/kg yeast selenium modulates the composition of T-lymphocyte subpopulations and increases total serum immunoglobulin concentrations in piglets (11). In addition to enhancing antioxidant capacity and immune function, selenium supplementation significantly affects the rumen microbiota of ruminants, primarily by increasing bacterial diversity, promoting the proliferation of beneficial microbes, and enhancing fiber degradation capacity (12–14). Alterations in rumen microbial fermentation patterns also influence the production and composition of volatile fatty acids (VFAs) (15). Variations in VFA concentrations directly reflect feed utilization efficiency, thereby influencing animal growth performance (16).

Current research on selenium (Se) has garnered significant attention in animal production; however, studies investigating the role of yeast selenium in Inner Mongolian white cashmere goats remain limited. This study aimed to evaluate the effects of dietary yeast selenium supplementation on growth performance, antioxidant and immune properties of serum and semen, and rumen microbiota in Inner Mongolian white cashmere goats. These findings provide a theoretical basis for the practical application of yeast selenium in the productivity of Inner Mongolian white cashmere goats.

## Materials and methods

### Test animals

Eighteen 2-year-old male Inner Mongolian cashmere goats with an initial average weight of 47.1 ± 1.1 kg were selected from the Inner Mongolia Autonomous Region Fleece Goat Breeding Farm, China (geographic coordinates between 97°10′ and 106°53′ East and 37°24′ and 42°47′ North) and randomly divided into two groups (*n* = 9). The selenium-enriched yeast used in this study is a commercial feed additive marketed as “Yeasts.” “Enriched selenium” (S29789, OriLeaf, Shanghai Yuanye Bio-Technology Co., Ltd., Shanghai, China) contained the following key components according to its nutritional profile: selenium (actual content: 2,231 mg/kg), moisture (2.9%), crude protein (53%), and crude ash (6.18%). In this study, the experimental cashmere goats were managed under a unified system, with grazing as the primary feeding method and concentrate supplementation as the secondary method. Each goat was provided 500 g of concentrate feed daily, divided into two portions of 250 g each—one in the morning before grazing and the other in the evening after returning from grazing. The concentrate feed composition was as follows: corn (35%), alfalfa pellets (35%), soybean meal (15%), and wheat bran (15%). Nutritional analysis of the concentrate feed showed the following results: crude protein (18.55% ± 3.71%), ash (11.43% ± 2.82%), fat (13.81% ± 2.65%), neutral detergent fiber (45.03% ± 7.48%), acid detergent fiber (15.64% ± 4.49%), and dry matter (92.38% ± 3.25%) contents. Each goat in the SE group was supplemented with 0.4 mg selenium from yeast daily (17). The experiment involved a combined grazing and supplemental feeding regime, with supplements provided daily at 8:00 a.m., followed by grazing at 9:00 a.m. The trial lasted for 35 days, including a 5-day pre-trial period and a 30-day formal experimental period. All goats had ad libitum access to feed and water throughout the study. All experimental methods used in this study were approved by the Animal Welfare and Ethics Committee of the Academy of Agricultural and Animal Husbandry Sciences of the Inner Mongolia Autonomous Region (Inner Mongolia, China; Ethical Approval No. 2025-0001).

### Sample collection

According to the experimental design and objectives, all goats in the CON and SE groups were weighed in the morning before grazing on the day the trial began and again in the morning before feeding on the day after the trial ended. Body weight data, as well as blood, semen, and rumen fluid samples, were collected. The specific sampling procedures and preservation methods are as follows.

#### Blood collection

Blood samples were collected via jugular venipuncture using 5 mL heparinized tubes, with approximately 10 mL of blood collected from each goat. Samples were stored on ice until centrifugation at 3,000 × g for 10 min at 4 °C to separate the serum. The serum was aliquoted, labeled, sealed, and stored at −20 °C. Blood samples from each male Inner Mongolian cashmere goat were collected again on the morning of the final day of the experiment using the same method.

#### Semen collection

Semen from male Inner Mongolian cashmere goats was collected using the electrical stimulation method (18). Fresh semen was examined under a microscope to determine sperm density and then diluted 5–10 times using a semen extender to ensure that all samples reached an appropriate sperm density. A portion of each fresh semen sample was processed using cryopreservation techniques and stored in liquid nitrogen (−196 °C) for long-term preservation to assess sperm motility. Another portion was centrifuged at 5,000 rpm for 10 min to separate the seminal plasma, which was then sealed and stored in liquid nitrogen (−196 °C). Upon returning to the laboratory, the seminal plasma was stored at −80 °C for long-term analysis of sperm quality-related parameters.

#### Collection of rumen fluid

The night before the Se supplementation period, male Inner Mongolian cashmere goats were subjected to a 12-h fast, during which feeding and drinking were prohibited. Early the next morning, before feeding, rumen fluid was collected from the control and experimental groups. Sterile containers and vacuum devices were used for sample collection. The vacuum device was activated when the gastric tube was inserted into the esophagus, and the first 20 mL of fluid was discarded to remove saliva mixed during the procedure. When the rumen fluid returned to its normal color, approximately 100 mL of fluid was collected and gently swirled several times to ensure adequate mixing. The liquid was filtered through four layers of sterile coarse cotton cloth to separate the solid impurities from the rumen fluid. For samples used for volatile fatty acid (VFA) determination, 10% metaphosphoric acid was added to effectively inhibit microbial growth and reduce the degradation of volatile fatty acids, thereby maintaining sample integrity. The rumen fluid was then transferred to 15 mL centrifuge tubes and 5 mL cryotubes and immediately frozen in liquid nitrogen. The 15 mL centrifuge tubes were used to determine pH, NH_3_-N, and VFAs, and 5 mL cryotubes were used for sequencing the 16S rRNA gene of microbial communities.

### Determination of antioxidant indexes, immunity indexes, and Se content

GSH-Px in blood and semen was determined by the Glutathione Peroxidase Activity Test Kit (HY-M0004); SOD was determined by the Superoxide Dismutase Test Kit (HY-M0001); and H_2_O_2_ was determined by H_2_O_2_ Test Kit (HY-M0019). Immunoglobulin A (IgA), immunoglobulin G (IgG), and immunoglobulin M (IgM) were determined using the Sheep Immunoglobulin IgG/A/M Detection Kit (HY-50094). All kits were obtained from Beijing Huaying Institute of Biotechnology, Beijing, China, and assays were performed according to the manufacturer’s instructions. Se levels in blood and semen were determined using an atomic absorption spectrometer (AAS, Shimadzu AA 670, equipped with a graphite furnace atomizer).

### Sperm viability tests

The method used to measure sperm motility in this study was based on a previously reported method (19). The procedure is briefly described as follows: in a dark environment, 0.5 μL of semen was mixed thoroughly with 5 μL of physiological saline and smeared onto a clean glass slide (37 °C). Sperm motility was analyzed using a computer-assisted sperm analysis (CASA) system. The system identifies all sperm in the captured images based on their morphology and records the motile sperm as viable. The viability rate was calculated as the proportion of motile sperm cells. The average was obtained from five randomly selected fields of view for each sample.

### Determination of rumen fermentation parameters

The rumen fluid was aspirated with a rubber-tipped burette into two drops in the sample disk of the pre-calibrated pH meter, the value was read, and the measurements were repeated three times after cleaning the sample disk with distilled water, and the average value was calculated. The rumen fluid samples were thawed and centrifuged at 5,400 r/min for 10 min, and 2 mL of the supernatant was added to the centrifuge tube. The NH_3_-N concentration in the samples was determined according to a previous method (20). The method and procedures for measuring VFAs in the rumen were adapted from the study by Lv et al. (21). The rumen fluid samples were thawed and centrifuged at 5,400 r/min for 10 min, and the contents of rumen fluid VFAs were determined using high-performance liquid chromatography (HPLC).

### Bioinformatics analysis

The collected rumen fluid was sent to Shanghai Ouyi Biomedical Technology Co., Ltd., and 343 F and 798 R primers were selected to amplify the V3–V4 variable region. The amplicons were sequenced using the Illumina NovaSeq platform to analyze the diversity of the rumen bacterial community and the composition structure of the rumen bacteria of cashmere goats using bioinformatics. Raw data obtained from the Illumina HiSeq (2500 PE250) sequencing platform were used for raw sequence quality control and splicing using Fastp software.^1^ Sequences were categorized into operational taxonomic units (OTUs) based on 97% similarity using the UPARSE software.^2^ Usearch software uses the Uchime algorithm to eliminate these chimeras. Each sequence was categorized and annotated at different levels (phylum and genus) using the Ribosomal Database Project (RDP), and the comparison database (SSU123) was compared with entries in the SILVA database. Alpha diversity (Shannon and Simpson) and richness (Observed_species and Chao) were analyzed using the parent software platform.^3^ Beta diversity analyses were performed using QIIME^4^ to compare differences in species diversity (microbial composition and structure) between samples.

### Analysis of microbial communities in relation to VFAs, ADGs, blood, semen immune properties, and antioxidant properties

Correlation analysis of eight differential microorganisms and indicators of ADG, rumen fluid VFA, and blood and semen antioxidant properties and immune performance obtained at the genus level between the SE and CON groups was performed using the OE online cloud platform.^5^

### Statistical analysis

An independent sample *t*-test using the Statistical Package for the Social Sciences (SPSS, version 26.0) was used to analyze the data on ADG, blood and semen antioxidant indices, immune indices, and rumen fluid VFAs of the velvet goats. Differences were considered statistically significant at *p* < 0.05.

## Results

### Selenium supplementation in rations improves growth performance of cashmere goats

Addition of yeast selenium to the diets of cashmere goats showed that the average daily weight gain of the SE group was significantly higher than that of the CON group at the end of the experiment (Table 1), despite the fact that the weight of the SE group did not show any significant upward trend compared to that of the CON group (Table 1), which proves that supplemental yeast selenium in the diets has a certain effect on the improvement of the growth performance of cashmere goats.

**Table 1:** Effect of selenium supplementation in feed on growth performance of cashmere goats.

Indexes	CON	SE	*p*-value
Post-test weight (kg)	47.22 ± 0.43	46.97 ± 0.36	0.673
Pre-test weight (kg)	49.58 ± 0.52	49.82 ± 0.40	0.729
Average daily weight gain (g)	78.88 ± 4.33	94.81 ± 5.55	0.038

CON, with no selenium added to diets, served as the negative control group (*n* = 9). SE, with yeast selenium added to diets (selenium added at a level of 0.4 mg/d), served as the test group (*n* = 9). Data are expressed as mean ± SEM, with *p* < 0.05 representing a significant difference.

### Selenium supplementation in rations improves antioxidant and immune properties of blood in cashmere goats

**Figure 1:**
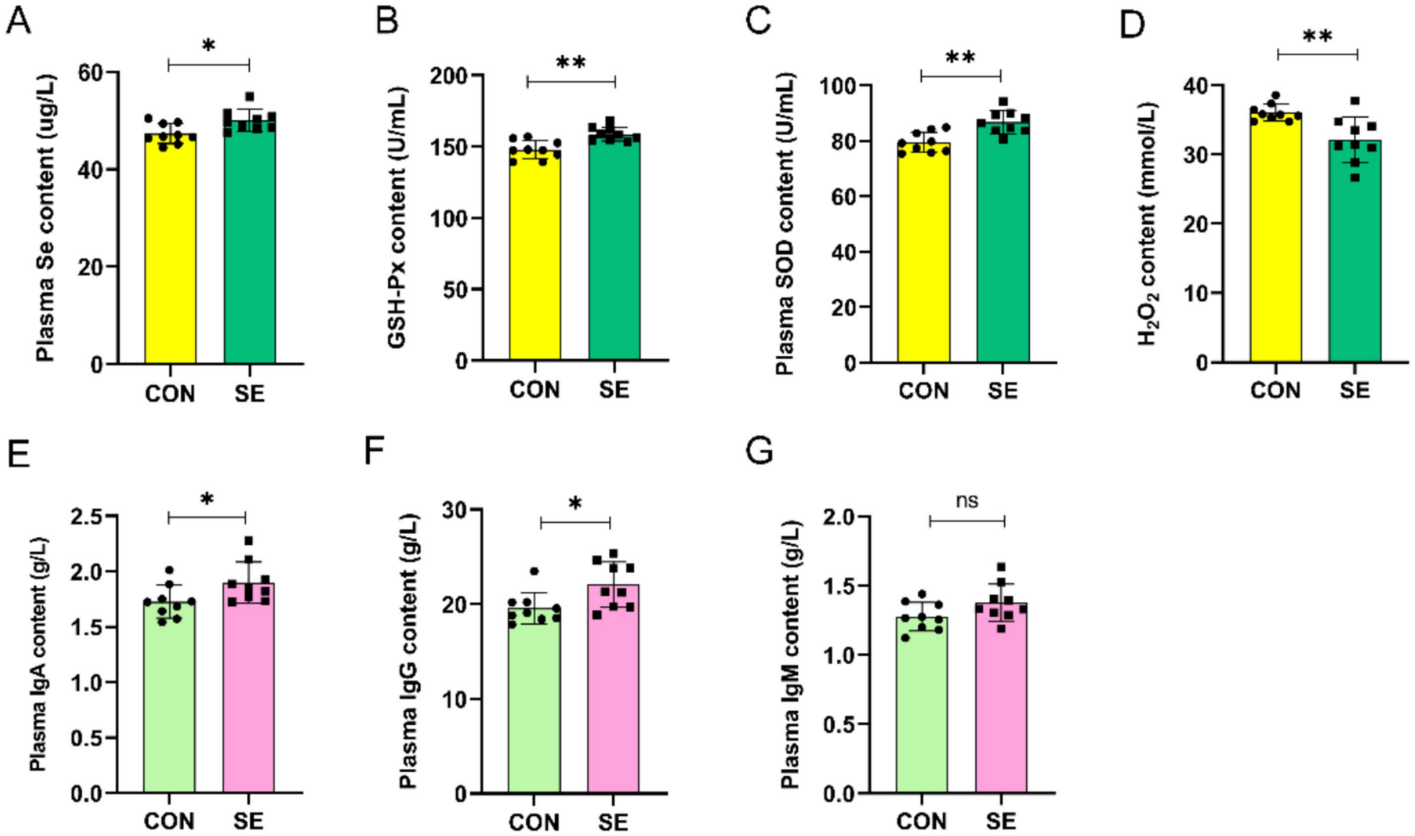
Effect of yeast selenium on blood antioxidant properties and immune performance in Cashmere Goats. **(A)** Plasma Se content. **(B)** Plasma GSH-Px content. **(C)** Plasma SOD content. **(D)** Plasma H₂O₂ content. **(E)** Plasma IgA content. **(F)** Plasma IgG content. **(G)** Plasma IgM content. CON: with no selenium added to diets served as the negative control group (*n* = 9). SE: with yeast selenium added to diets (selenium added at a level of 0.4 mg/d) served as the test group (*n* = 9). GSH-Px: glutathione peroxidase; SOD: superoxide dismutase; H₂O₂: hydrogen peroxide; IgA: immunoglobulin A; IgG: immunoglobulin G; IgM: immunoglobulin M. *p < 0.05, **p < 0.01, ***p < 0.001, ^ns^*p* > 0.05.

The addition of selenium to the ration significantly increased the level of Se in blood compared to those in the CON group (Figure 1A). At the same time, blood levels of GSH-Px and SOD were also significantly increased (Figures 1B,C), whereas blood levels of H_2_O_2_ were significantly lower than those in the CON group (Figure 1D). Supplementation of Se in the diet also improved the immune properties of the blood of cashmere goats, and compared with the CON group, there was a significant increase in the IgA and IgG content of the blood in the SE group, with no significant effect on the IgM content (Figures 1E–G).

### Selenium supplementation in the ration to improve the antioxidant and immune properties of the spermatozoa of cashmere goats

**Figure 2:**
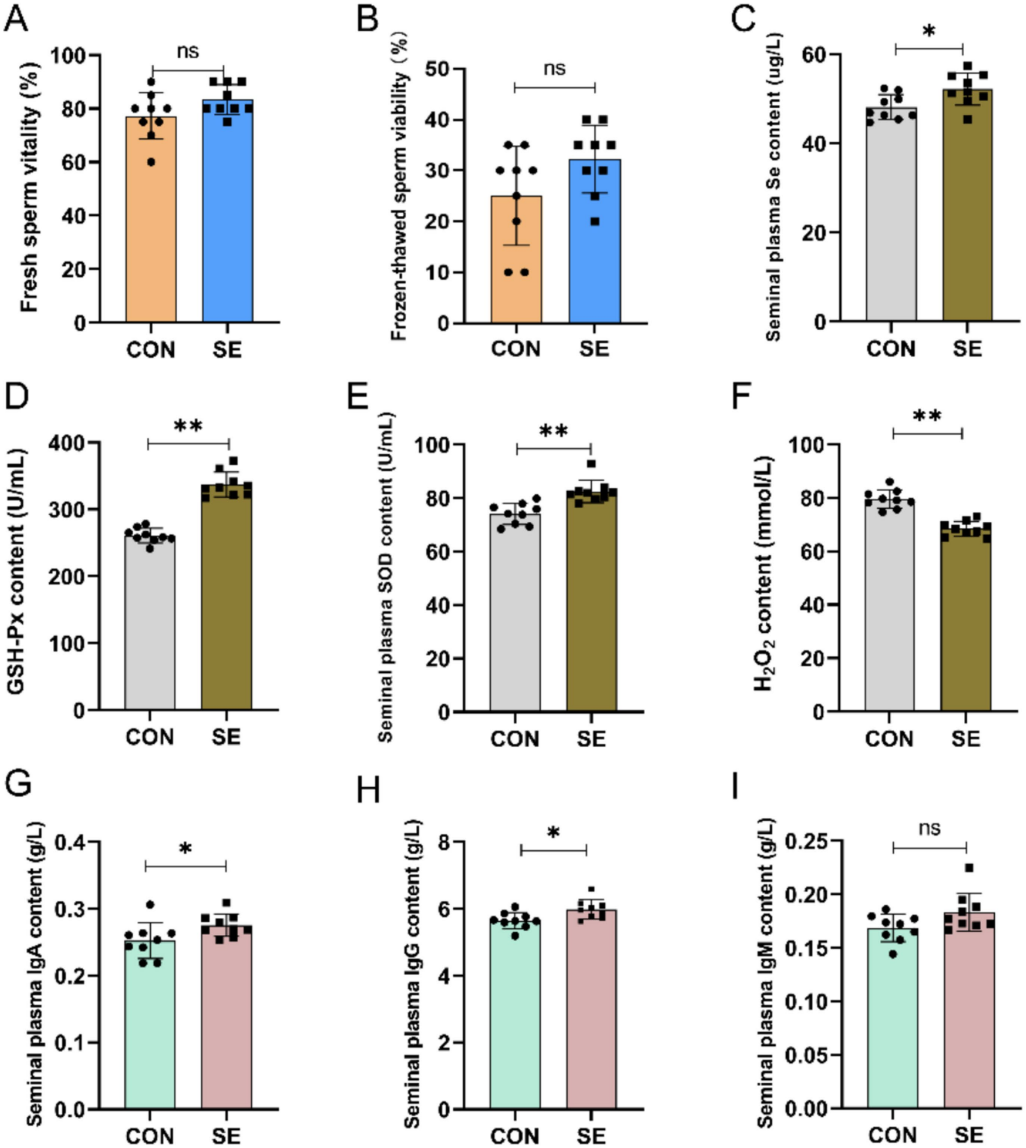
Effect of yeast selenium on spermatozoa viability, antioxidant properties and immune performance in Cashmere Goats. **(A)** Fresh sperm vitality. **(B)** Frozen-thawed sperm viability. **(C)** Seminal plasma Se content. **(D)** Seminal plasma GSH-Px content. **(E)** Seminal plasma SOD content. **(F)** Seminal plasma H₂O₂ content. **(G)** Seminal plasma IgA content. **(H)** Seminal plasma IgG content. **(I)** Seminal plasma IgM content. CON: with no selenium added to diets served as the negative control group (*n* = 9). SE: with yeast selenium added to diets (selenium added at a level of 0.4 mg/d) served as the test group ((*n* = 9). GSH-Px: glutathione peroxidase; SOD: superoxide dismutase; H₂O₂: hydrogen peroxide; IgA: immunoglobulin A; IgG: immunoglobulin G; IgM: immunoglobulin M. *p < < 0.05, **p < < 0.01, ***p < 0.001, ^ns^*p* > 0.05.

Compared with the CON group, supplemental feeding of Se had a certain effect on the improvement of sperm viability before and after freeze-thawing in cashmere goats, but the difference was not significant (Figures 2A,B). By supplementing yeast selenium, the content of Se in the semen of the SE group was also significantly improved compared to that of the CON group (Figure 2C). Meanwhile, supplemental feeding of Se also improved the antioxidant and immune properties of semen, with significantly higher levels of GSH-Px and SOD (Figures 2D,E) and significantly lower levels of H_2_O_2_ (Figure 2F) in the semen of the SE group compared with the CON group. Immunoglobulin IgA and IgG levels were also significantly increased in the SE group, and IgM levels were not significantly affected (Figure 2G–I).

### Effect of selenium supplementation in rations on rumen fluid fermentation parameters

As shown in Table 2, supplementation with yeast selenium significantly increased the NH_3_-N content of the rumen fluid of cashmere goats (*p* < 0.05). Compared with the CON group, the content of propionic acid in the rumen fluid of velvet goats in the SE group increased, and the content of acetic acid and n-butyric acid decreased; however, the differences were not significant (*p* > 0.05).

**Table 2:** Effect of yeast selenium on rumen fermentation parameters in cashmere goats.

Items	CON	SE	*p*-value
pH	7.13 ± 0.12	7.44 ± 0.07	0.050
NH_3_-N (mg/dL)	9.93 ± 0.33	11.70 ± 0.37	0.003
Acetate (mmol/L)	44.38 ± 3.66	37.88 ± 1.06	0.122
Propionate (mmol/L)	19.41 ± 1.49	23.14 ± 1.38	0.085
Butyrate (mmol/L)	2.47 ± 0.27	2.25 ± 0.10	0.472

CON, with no selenium added to diets, served as the negative control group (*n* = 9). SE, with yeast selenium added to diets (selenium added at a level of 0.4 mg/d), served as the test group (*n* = 9). NH_3_-N, ammoniacal nitrogen. Data are expressed as mean ± SEM, with *p* < 0.05 representing a significant difference.

### Sequencing of the rumen microbiota

**Figure 3:**
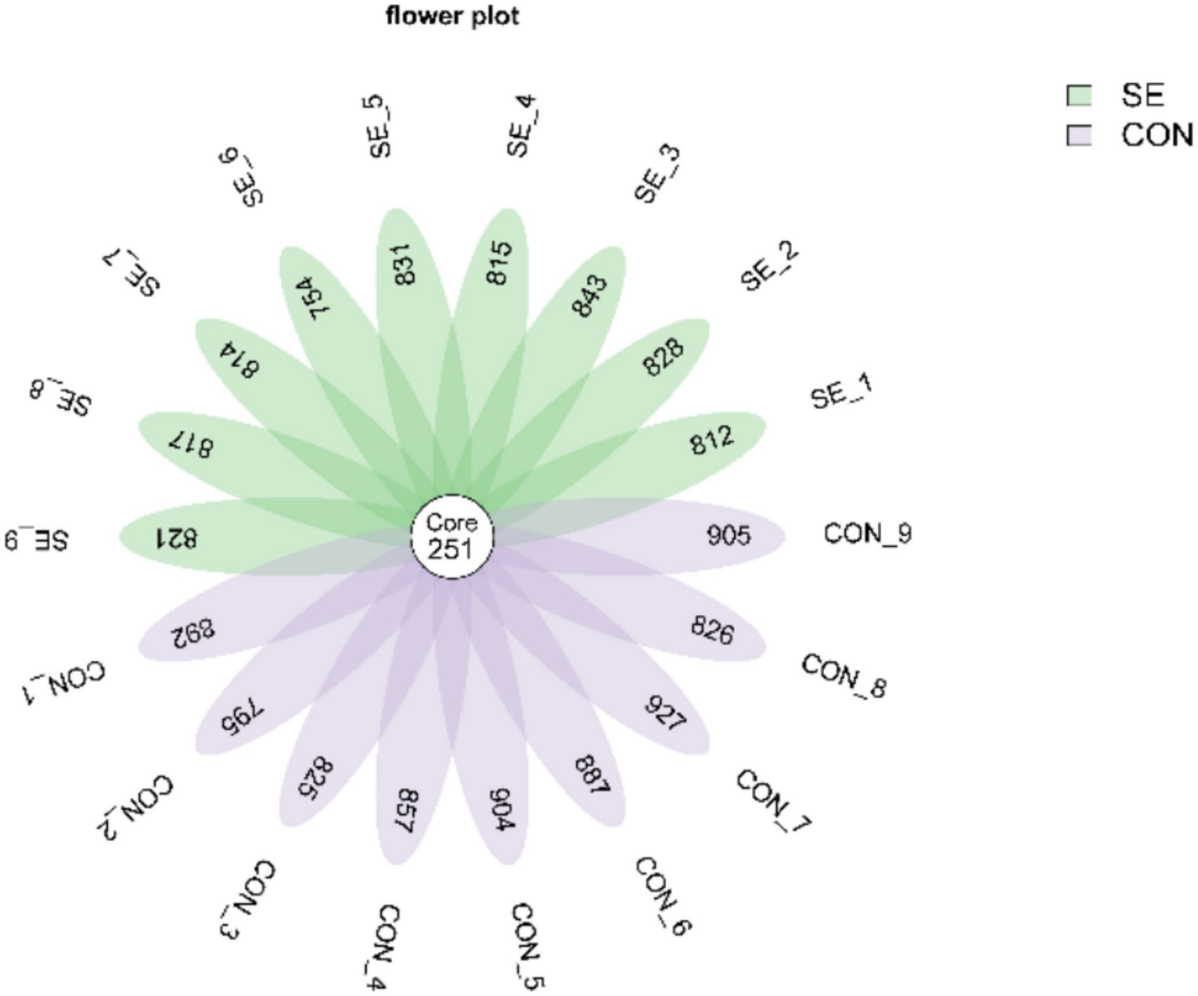
Venn plot of ASV distribution. CON, with no selenium added to diets served as the negative control group (*n* = 9). SE, with yeast selenium added to diets (selenium added at a level of 0.4 mg/d), served as the test group (*n* = 9).

Raw downstream data from 18 samples were analyzed. The data volume of clean tags after sequencing downstream raw read quality control filtering was distributed between 56,706 and 62,210. Clean tags, after removing chimeras to obtain the data volume of valid tags, were distributed between 52,451 and 58,170. Further de-emphasis of the sequences was performed to obtain each sample with a feature sequence of more than 1,005–1,178 items, defined as amplicon sequence variants (ASVs). De-duplication of the ASV sequences obtained for all samples revealed that there were 251 ASVs common to all samples, with a total of 2,938 ASV sequences obtained for the CON group and 2,901 ASV sequences obtained for the SE group (Figure 3).

### Changes in the rumen microbial community

**Figure 4:**
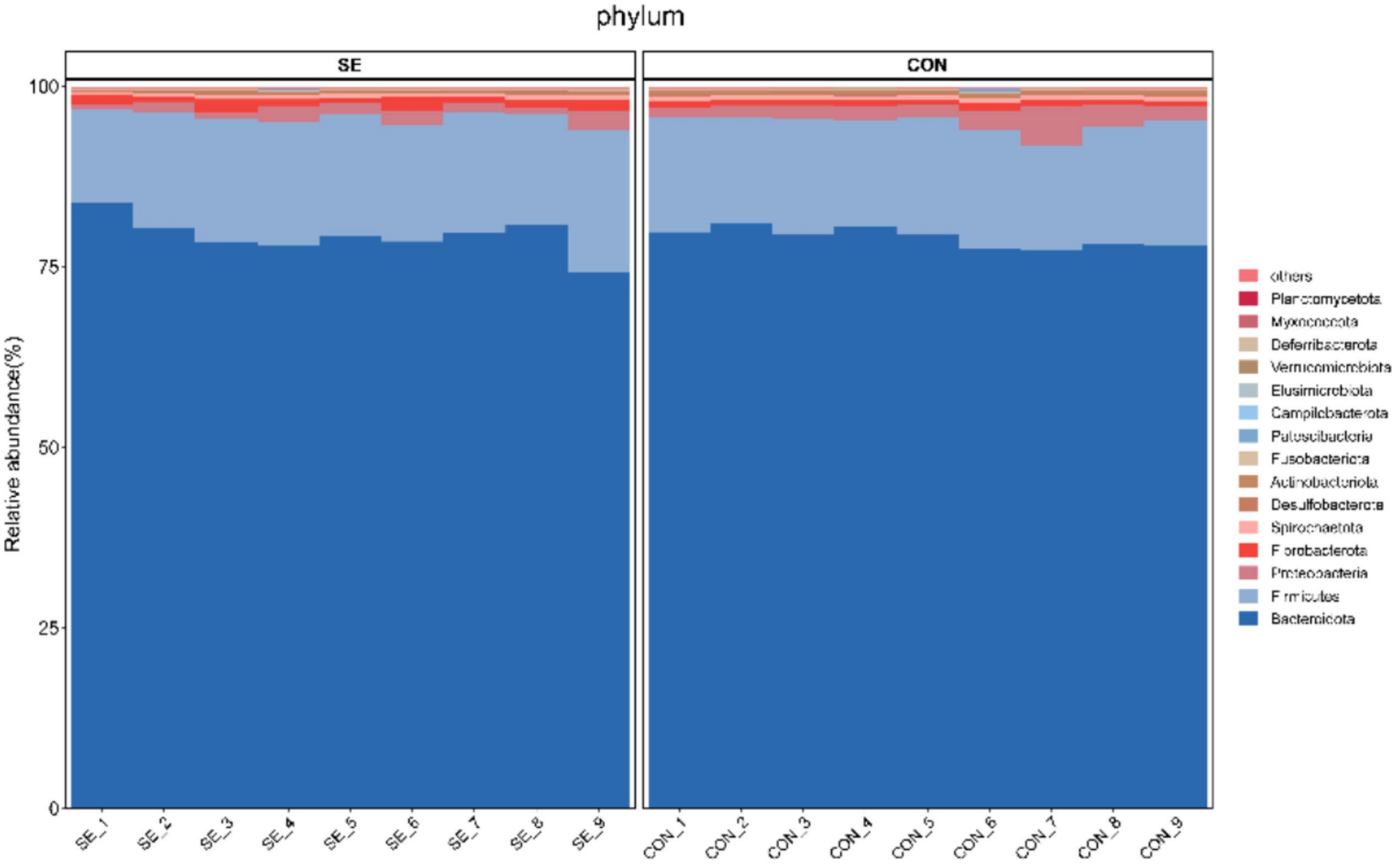
Histogram of phylum-level community structures. CON, with no selenium added to diets served as the negative control group (*n* = 9). SE, with yeast selenium added to diets (selenium added at a level of 0.4 mg/d), served as the test group (*n* = 9).

**Figure 5:**
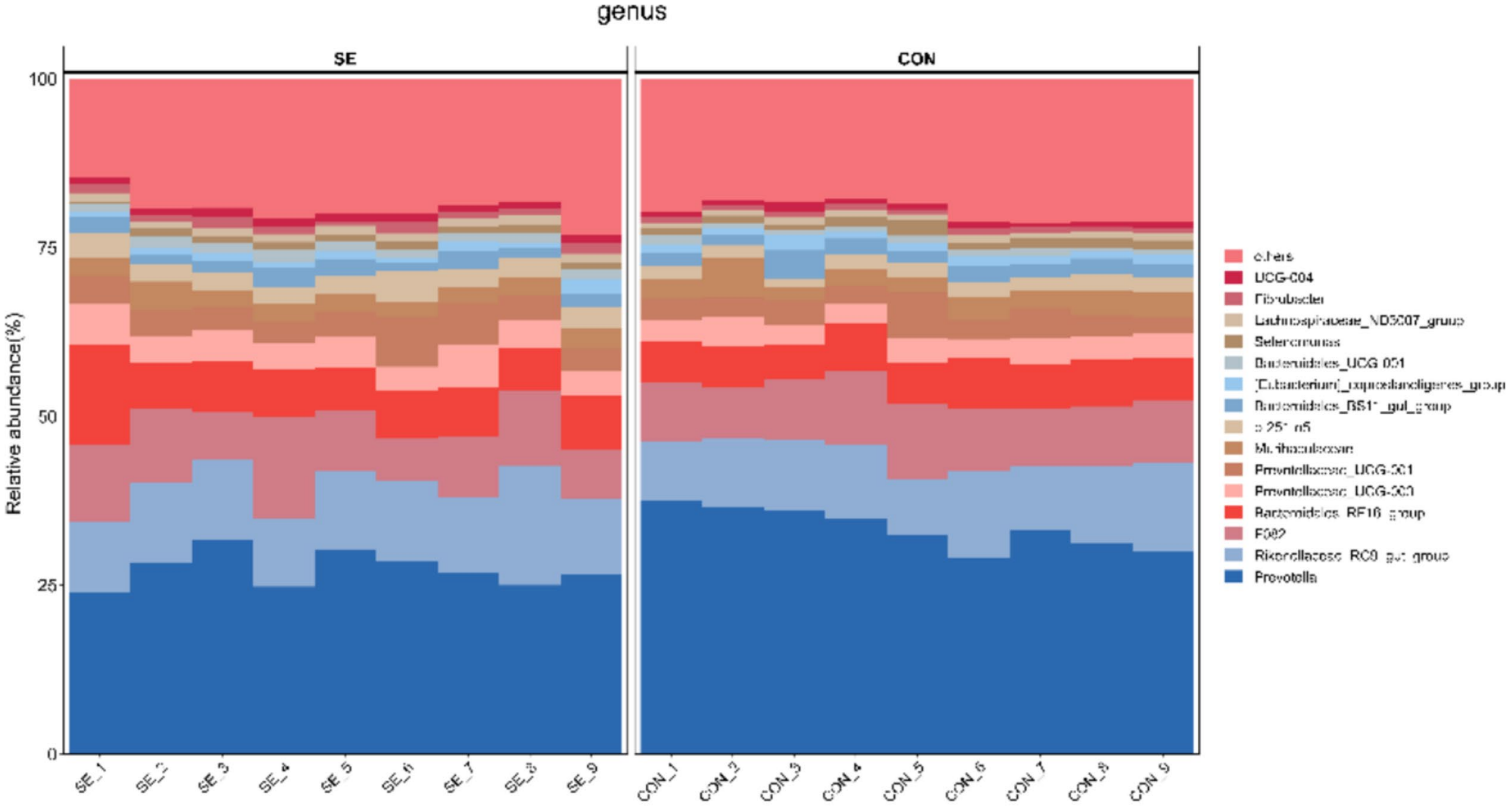
Histogram of community structure at the genus level. CON, with no selenium added to diets served as the negative control group (*n* = 9). SE, with yeast selenium added to diets (selenium added at a level of 0.4 mg/d), served as the test group (*n* = 9).

Phylum-level statistics showed that Bacteroidota and Firmicutes were the dominant phyla, with Bacteroidota accounting for 74.22–83.89% and Firmicutes accounting for 13.01–19.84% (Figure 4). Genus-level statistics showed that *Prevotella*, *Rikenellaceae_RC9_gut_group*, *F082*, and *Bacteroidales_RF16_group* were the dominant genera, with *Prevotella* accounting for 23.91–37.55%, *Rikenellaceae_RC9_gut_group* 8.33–17.73%, *F082* 6.38–15.03%, and *Bacteroidales_RF16_group* 5.05–14.97% (Figure 5).

### Alpha diversity of the rumen microbiota

The results of the alpha diversity analysis (Table 3) showed that the observed and Chao indices were significantly reduced, and the Shannon and Simpson indices did not change significantly after Se supplementation. This indicated that the abundance of the rumen microbial community was significantly reduced after Se supplementation in the feed, whereas the diversity of the microbial community did not change significantly.

**Table 3:** Effect of yeast selenium on the alpha diversity index in cashmere goats.

Indexes	CON	SE	*p*-value
Shannon	9.01 ± 0.19	8.91 ± 0.28	0.456
Simpson	0.99 ± 0.002	0.99 ± 0.003	0.809
Observed_species	1117.89 ± 41.91	1065.30 ± 23.61	0.006
Chao	1120.68 ± 42.60	1061.27 ± 23.46	0.006

CON, with no selenium added to diets served as the negative control group (*n* = 9). SE, with yeast selenium added to diets (selenium added at a level of 0.4 mg/d), served as the test group (*n* = 9). Data are expressed as mean ± SEM, with *p* < 0.05 representing a significant difference.

### Beta diversity of the rumen microbiota

**Figure 6:**
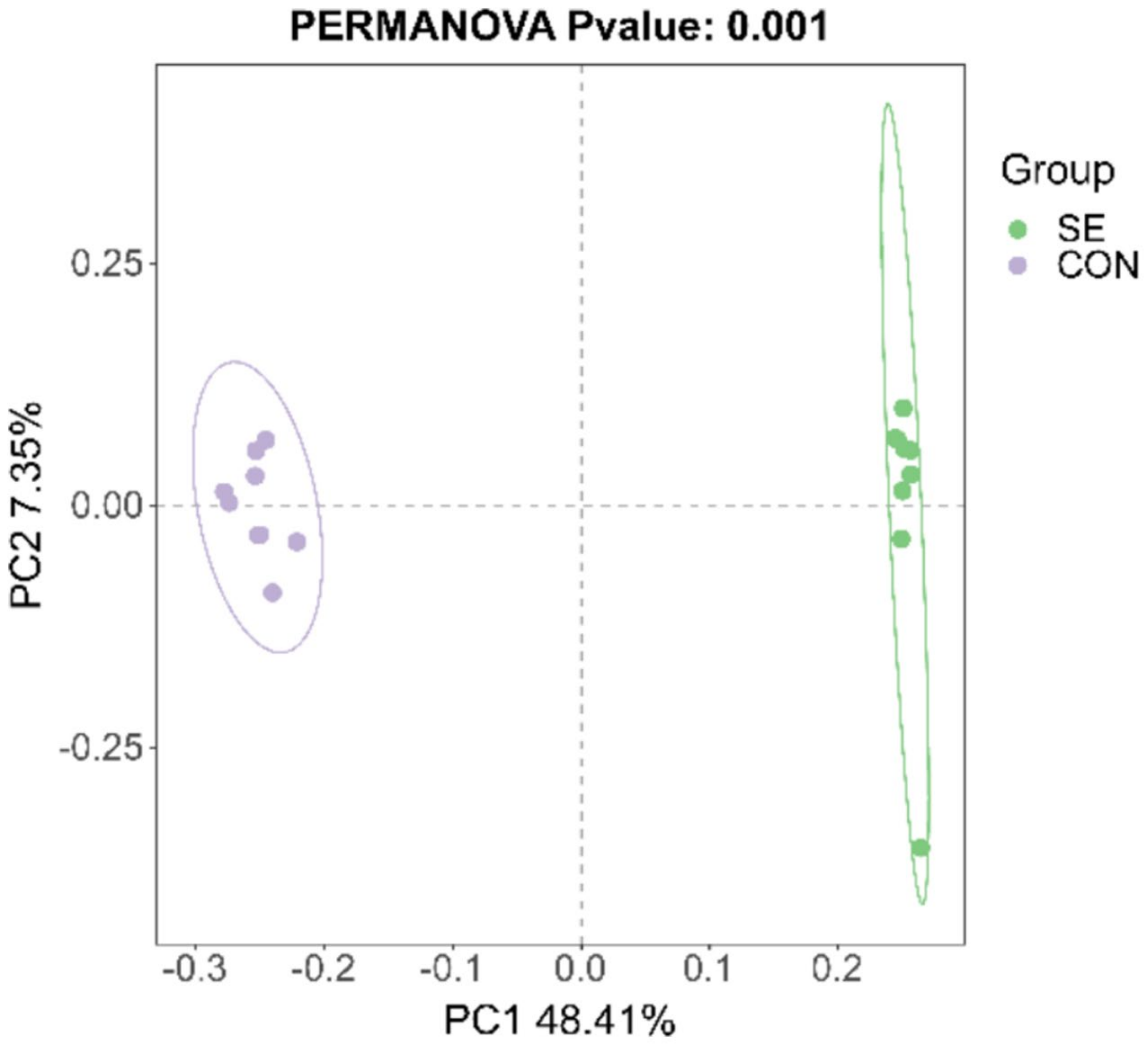
PCoA statistics. CON, with no selenium added to diets served as the negative control group (*n* = 9). SE, with yeast selenium added to diets (selenium added at a level of 0.4 mg/d), served as the test group (*n* = 9).

The PCoA results showed that the samples were well clustered within the CON and SE groups and that the within-group differences were smaller than the between-group differences (Figure 6). Significant differences in beta diversity were found using the Bray–Curtis distance algorithm (*p* = 0.001), and Adonis statistics showed an *R*^2^ of 0.55.

### Analysis of differences in microbial flora

**Figure 7:**
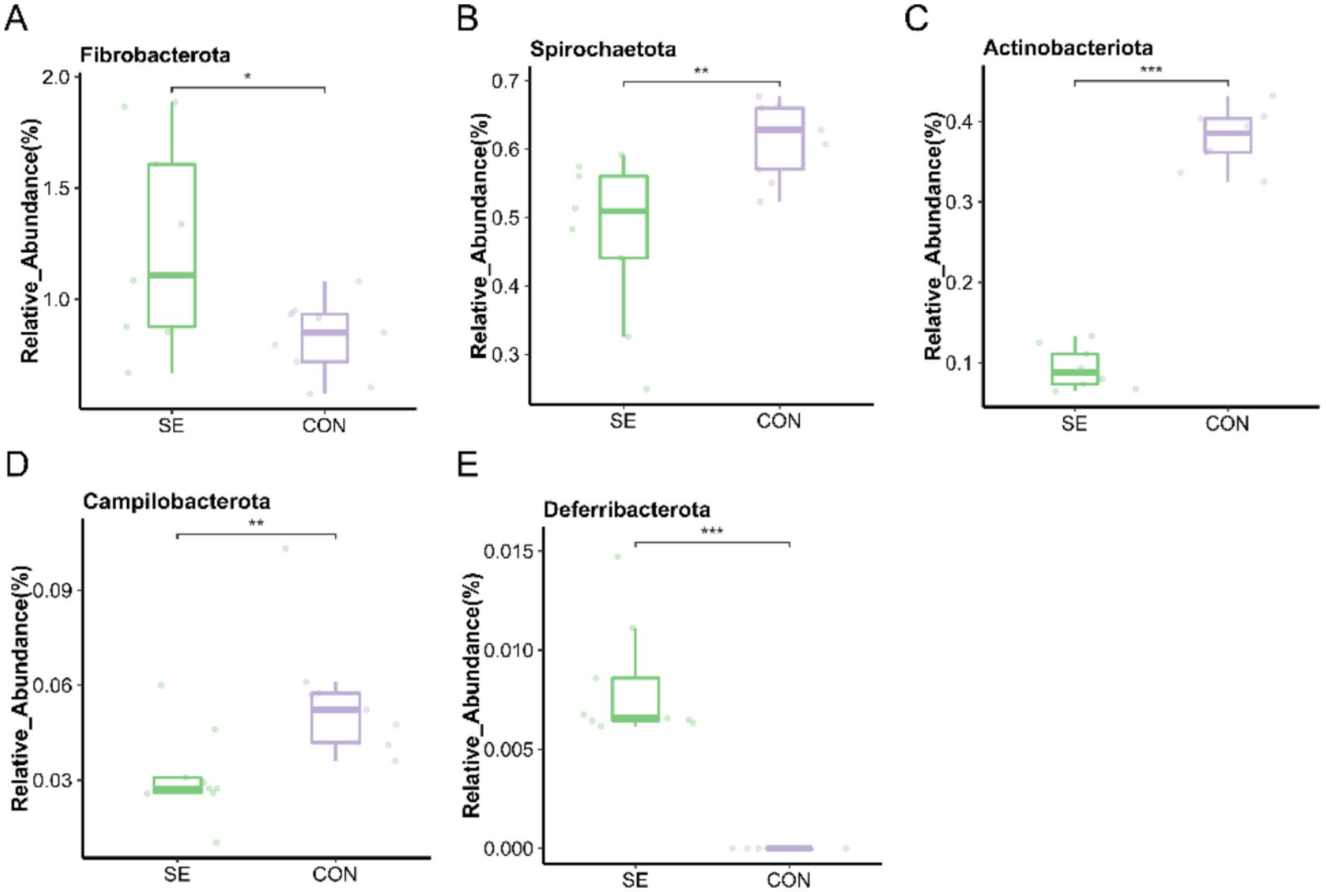
Phylum level individual bacterial differential species abundance map. **(A)** Fibrobacterota relative abundance. **(B)** Spirochaetota relative abundance. **(C)** Actinobacteriota relative abundance. **(D)** Campilobacterota relative abundance. **(E)** Deferribacterota relative abundance. CON: with no selenium added to diets served as the negative control group (*n* = 9). SE: with yeast selenium added to diets (selenium added at a level of 0.4 mg/d) served as the test group (*n* = 9). *p < 0.05, **p < 0.01, ***p < 0.001.

At the phylum level, the microorganisms with the top 15 relative abundances were statistically analyzed, and five differential microorganisms were identified. Compared with the CON group, the relative abundances of *Fibrobacterota* and *Deferribacterota* were significantly higher (*p* < 0.05), and those of *Spirochaetota*, *Actinobacteriota*, and *Campilobacterota* were significantly lower (*p* < 0.05) after the addition of Se (Figure 7).

**Figure 8:**
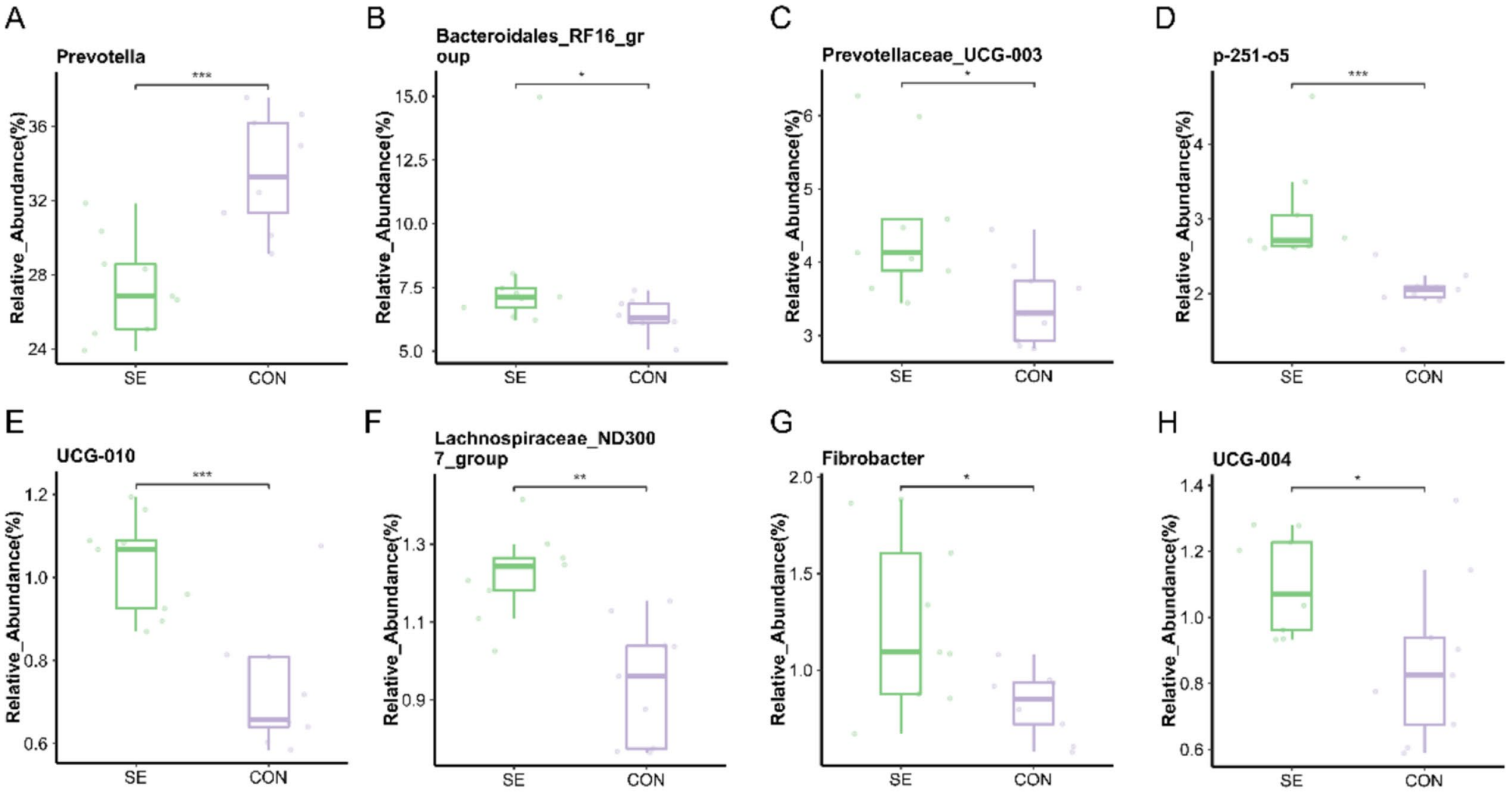
Genus level individual bacterial differential species abundance map. **(A)** Prevotella relative abundance. **(B)** Bacteroidales_RF16_group relative abundance. **(C)** Prevotellaceae_UCG-003 relative abundance. **(D)** p-251-o5 relative abundance. **(E)** UCG-010 relative abundance. **(F)** Lachnospiraceae_ND3007_group relative abundance. **(G)** Fibrobacter relative abundance. **(H)** UCG-004 relative abundance. CON: with no selenium added to diets served as the negative control group (*n* = 9). SE: with yeast selenium added to diets (selenium added at a level of 0.4 mg/d) served as the test group (*n* = 9). *p < 0.05, **p < 0.01, ***p < 0.001.

At the genus level, the microorganisms with the top 15 relative abundances were statistically analyzed, and eight differential microorganisms were found. Compared with the CON group, the relative abundance of *Prevotella* was significantly reduced (*p* < 0.05) with the addition of Se, and the relative abundance of *Bacteroidales_RF16_group*, *Prevotellaceae_UCG-003*, *p-251-o5*, *UCG-010*, *Lachnospiraceae_ND3007_group*, *Fibrobacter*, and *UCG-004* was significantly higher (*p* < 0.05) (Figure 8).

### Correlation analysis of microbial communities with VFAs, ADGs, blood, semen immune properties, and antioxidant properties

**Figure 9:**
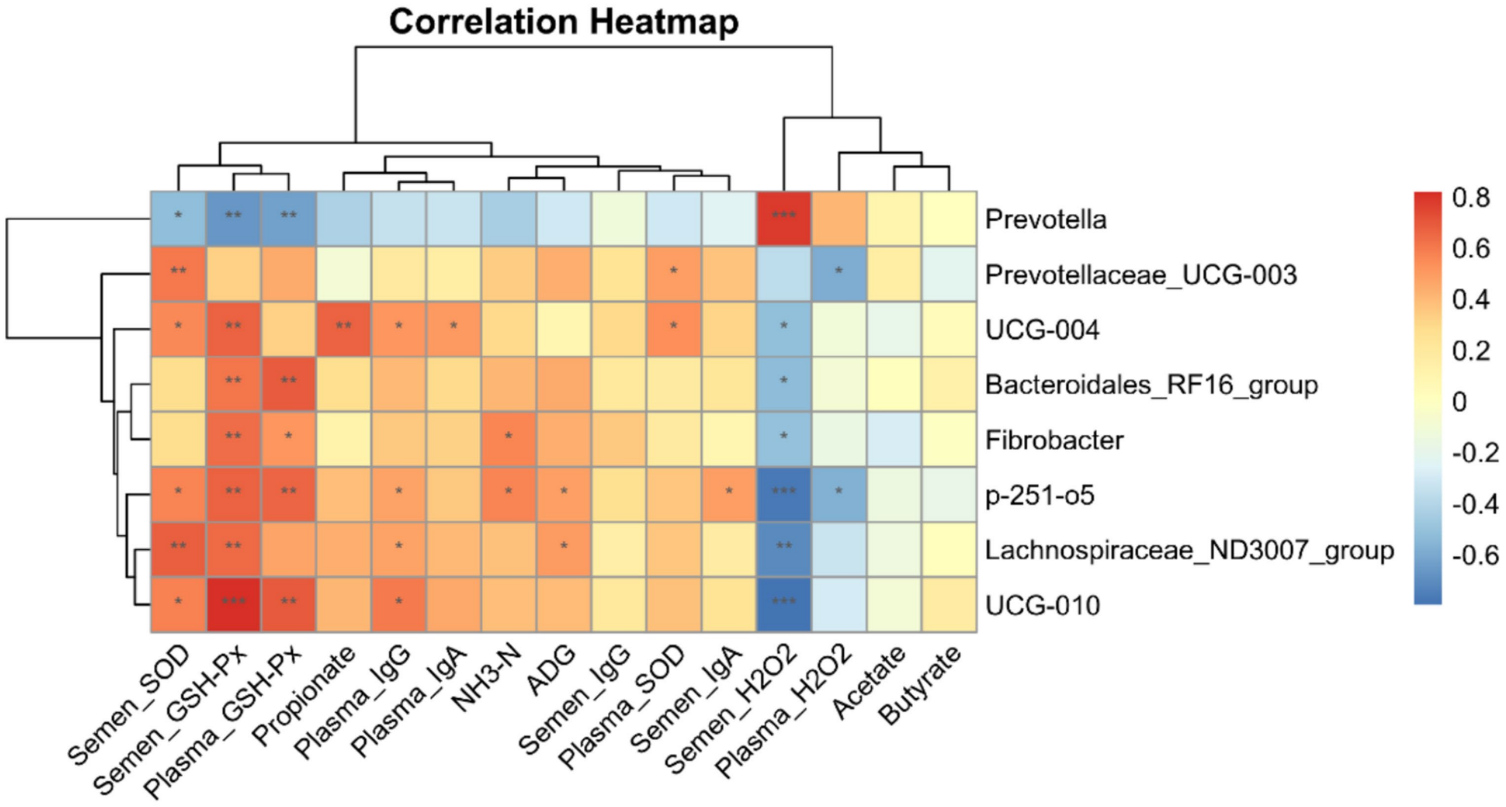
Correlation heatmap analysis. Correlation analysis of eight differential microorganisms and indicators of ADG, rumen fluid VFA, and blood and semen antioxidant properties and immune performance obtained at the genus level between the SE and CON groups. CON, with no selenium added to diets served as the negative control group (*n* = 9). SE, with yeast selenium added to diets (selenium added at a level of 0.4 mg/d), served as the test group (*n* = 9). ADG, average daily weight gain; VFA, volatile fatty acids; GSH-Px, glutathione peroxidase; SOD, superoxide dismutase; H₂O₂, hydrogen peroxide; IgA, immunoglobulin A; IgG, immunoglobulin G; IgM, immunoglobulin M. ^*^p < 0.05, ^**^p < 0.01, and ^***^p < 0.001.

The results of the correlation analysis of the differences in microorganisms at the genus level between the SE and CON groups and the indices of ADG and rumen fluid VFA, as well as the antioxidant and immune properties of blood and semen of the downy goats, are shown in Figure 9. There was a significant positive correlation between the H_2_O_2_ enzyme content in the semen and *Prevotella* and a negative correlation with *UCG-004*, *Bacteroidales_RF16_group*, *p-251-o5*, *UCG-010*, *Lachnospiraceae_ND3007_group*, and *Fibrobacter*. The GSH-Px and SOD levels in the semen were significantly and positively correlated with *UCG-004*, *UCG-010*, *p-251-o5*, and *Lachnospiraceae_ND3007_group*. In addition, *p-251-o5* and *Fibrobacter* were positively correlated with NH_3_-N content in the rumen of cashmere goats, and *p-251-o5* and *Lachnospiraceae_ND3007_group* were significantly positively correlated with ADG in cashmere goats. Among the differential microorganisms, *UCG-004*, *UCG-010*, and *p-251-o5* were positively correlated with IgG levels in the blood.

## Discussion

As an essential trace element for ruminants, selenium plays a crucial regulatory role in immunity, antioxidation, growth and development, and reproduction (22). Selenium deficiency can result in reduced performance, impaired immunity, and a range of selenium deficiency-related diseases (23, 24). Therefore, investigating dietary selenium supplementation in ruminant nutrition is essential for promoting healthy breeding and overall productivity.

Selenoproteins derived from yeast selenium enhance animal productivity primarily by improving systemic antioxidant and immune functions (23). Supplementation with yeast selenium increased the blood total antioxidant capacity (T-AOC) and glutathione peroxidase (GSH-Px) activity in lambs by 18.1 and 17.6%, respectively (25). T-AOC directly reflects the body’s overall antioxidant status (26), and GSH-Px protects hemoglobin and fatty acids from oxidation, scavenges free radicals, and prevents cellular membrane damage, thereby enhancing the antioxidant defense (27). In the present study, yeast selenium supplementation increased blood GSH-Px levels and decreased H₂O₂ content in Inner Mongolian White cashmere goats, confirming its effectiveness in enhancing antioxidant capacity in this breed. Blood immunoglobulins A (IgA), G (IgG), and M (IgM) are critical components of the immune system (28). Supplementing sow diets with 1.2 mg/kg yeast selenium increased colostrum IgM levels by 35% and mature milk IgA levels by 28%. These were transferred to piglets via lactation, resulting in a 30% increase in plasma IgG levels and reduced diarrhea and mortality rates (29). Supplementation with 0.36 mg/kg yeast selenium in fattening pig diets upregulated the expression of genes related to innate immunity, indicating an enhanced cellular immune response (30). In the present study, yeast selenium supplementation significantly increased blood IgA and IgG levels in velvet goats, demonstrating its role in enhancing the immune performance of cashmere goats.

Selenium mitigates oxidative damage to sperm cell membranes in male animals by suppressing lipid peroxidation, thereby preserving membrane integrity and enhancing sperm quality (31, 32). For example, selenium influences the proliferation and apoptosis of sheep Leydig cells by modulating oxidative stress and regulating the expression of cell cycle- and apoptosis-related genes (33). In the present study, yeast selenium supplementation enhanced sperm viability in cashmere goats both before and after freeze–thawing. Yeast selenium supplementation also significantly increased the activities of GSH-Px and SOD while reducing H₂O₂ levels in the semen of cashmere goats. These findings indicate that yeast selenium supplementation alleviates oxidative damage during sperm cryopreservation and positively contributes to the reproductive performance of cashmere goats.

Dietary selenium can be metabolized by rumen microorganisms, potentially influencing rumen microbial activity and fermentation. Several studies have demonstrated that organic selenium improves rumen fermentation parameters (15, 34). Selenium supplementation has been shown to increase total volatile fatty acid (TVFA) concentrations in the rumen, shifting fermentation patterns toward propionate production and enhancing rumen microbial activity, thereby promoting nutrient fermentation (35–37). These findings are consistent with the increased propionic acid concentration observed in the yeast selenium-supplemented group in the present study. Ammonia nitrogen (NH₃-N) is the sole direct source of microbial nitrogen in the rumen, and its concentration reflects the rate of microbial degradation and utilization of nitrogenous compounds (38, 39). In this study, dietary supplementation with yeast selenium significantly increased the NH₃-N concentration in rumen fluid, indicating enhanced rumen microbial activity and improved capacity for microbial protein synthesis in cashmere goats.

In the rumen, thick-walled Bacteroidota are Gram-positive bacteria that primarily produce propionate and butyrate, whereas Bacteroidota anisoplia are Gram-negative bacteria that specialize in fiber degradation and volatile fatty acid (VFA) production (40). Bacteroidota anisoplia is a major fiber-degrading group in the rumen, capable of hydrolyzing complex polysaccharides, such as cellulose and hemicellulose, and producing short-chain fatty acids (SCFAs), particularly acetic and propionic acids, which supply energy to the host (38). *Prevotella* spp. are core members of the rumen microbiota, specializing in starch and soluble sugar degradation and protein and peptide metabolism (41). In the present study, the relative abundances of Fibrobacterota and Deferribacterota were significantly elevated at the portal level, possibly due to the dietary selenium supplementation. Changes in the rumen microbiota can affect multiple physiological functions in ruminants. Accordingly, we analyzed the correlations between differential microbial taxa and average daily gain (ADG), ruminal VFA concentrations, and antioxidant and immune parameters in the blood and semen of downy goats. We found that the abundances of UCG-004, p-251-o5, and UCG-010 were positively correlated with glutathione peroxidase (GSH-Px) and superoxide dismutase (SOD) levels in semen. Yeast selenium may directly regulate GSH-Px and SOD levels to enhance antioxidant defenses while modulating rumen microbial populations, thereby improving overall antioxidant activity, sperm motility, and reproductive performance in goats.

## Conclusion

Supplemental feeding of selenium yeast can improve the growth and reproductive performance and antioxidant and immune properties of Inner Mongolian cashmere goats. Meanwhile, the increase in the abundance of *UCG-004*, *UCG-010*, and *p-251-o5* in the rumen induced by selenium yeast supplementation was associated with improved antioxidant and immune performance in cashmere goats.

## Data Availability

The microbial sequences in our manuscript have been deposited in the Sequence Read Archive (SRA) of the NCBI, Accession Nos. SAMN48999589–SAMN48999606.

## Footnotes

^1^ https://ccb.jhu.edu/software/FLASH/index.shtml

^2^ http://drive5.com/uparse/

^3^ https://mothur.org/wiki/download_mothur/

^4^ http://qiime.org/install/index.html

^5^ https://cloud.oebiotech.com/#/bio/tools
